# Lifestyle intervention to prevent excessive maternal weight gain: mother and infant follow-up at 12 months postpartum

**DOI:** 10.1186/s12884-015-0701-2

**Published:** 2015-10-15

**Authors:** Kathrin Rauh, Julia Günther, Julia Kunath, Lynne Stecher, Hans Hauner

**Affiliations:** ZIEL – Research Centre for Nutrition and Food Sciences, Technische Universität München, Freising-Weihenstephan, Germany; Competence Centre for Nutrition (KErn), Freising, Germany; Else Kröner-Fresenius-Center for Nutritional Medicine, Chair of Nutritional Medicine, Technische Universität München, Freising-Weihenstephan, Germany; Else Kröner-Fresenius-Centre for Nutritional Medicine, Klinikum rechts der Isar, Technische Universität München, Munich, Germany

**Keywords:** Gestational weight gain (GWG), Lifestyle intervention, Pregnancy, Postpartum weight retention, Obesity prevention and management, Childhood adiposity

## Abstract

**Background:**

Excessive gestational weight gain (GWG) is associated with elevated weight retention in mothers and might be related to adiposity of their offspring. Little is known if lifestyle intervention during pregnancy has beneficial effects for mothers and children beyond gestation.

**Methods:**

A cluster-randomized controlled intervention trial was performed with 250 pregnant women in 8 gynaecological practices. Lifestyle intervention was carried out twice with individual counselling sessions on nutrition, physical activity and weight monitoring. Participants in the control group received routine prenatal care and an information leaflet. Follow-up data of women and their offspring were collected one year postpartum (pp) by phone call and/or via e-mail using a structured questionnaire. Maternal weight retention at 12 months pp and weight development of the children in their first year of life was compared between groups using linear regression. The association between energy and macronutrient intake during pregnancy with maternal weight retention and children weight development was also assessed.

**Results:**

The intervention resulted in a trend towards lower mean weight retention 12 months pp (0.2 vs. 0.8 kg), but was not statistically significant (*p* = 0.321). Among women receiving lifestyle counselling, only 8 % retained more than 5 kg weight while 17 % in the control group retained >5 kg (OR: 0.40 (95 % CI: 0.16, 0.97)). For the whole study cohort, an association between higher GWG and increased 12 month weight retention was found (0.4 kg weight retention per 1 kg increase in GWG, *p* < 0.001). Weight development of the infants did not differ between groups in the first months after birth. At the 10^th^–12^th^ month weight measurement, infants born to mothers in the intervention group tended towards lower body weights. Both energy intake and macronutrient composition of the diet during pregnancy did not affect maternal weight retention and weight development of the infants.

**Conclusions:**

Lifestyle counselling during pregnancy to avoid GWG had a rather modest effect on maternal pp weight retention and weight development of the infants. However, larger intervention studies and longer follow-up are required to be able to draw definite conclusions.

**Trial registration:**

German Clinical Trials Register DRKS00003801.

**Electronic supplementary material:**

The online version of this article (doi:10.1186/s12884-015-0701-2) contains supplementary material, which is available to authorized users.

## Background

Over the last decades, the global prevalence of obesity has increased considerably [1]. A rise in the prevalence of obesity has also been observed in Germany where more than 23 % of both adult men and women are obese [2]. Especially among young adults aged 25–34 years, including women of reproductive age, an alarming rise in the prevalence of obesity has been reported [3]. Hence, this trend is relevant in the area of pregnancy and childbirth. Maternal overweight and obesity have become a major health problem bearing risks for both mothers and children [4–9].

In addition to obesity, excessive gestational weight gain (GWG) has been identified as a risk factor for pregnancy complications [10–16]. Additionally, the amount of weight gain during pregnancy may have a strong impact on maternal weight retention [17–20] and seems to be a predictive factor for the long-term development of obesity in the mother [18, 21–23]. Excessive GWG can also affect foetal growth and is associated with an increased proportion of large for gestational age (LGA) newborns [16, 24–26]. In addition, excessive gestational weight gain was reported to be associated with childhood obesity [27–35] and obesity in later stages of life [36–40].

Lifestyle intervention during pregnancy seems to be a promising strategy to prevent excessive GWG [41–45]. We have previously reported the first results of a lifestyle intervention in pregnancy to reduce the rate of excessive gestational weight gain (FeLIPO trial) [46]. The lifestyle intervention resulted in a lower proportion of women exceeding the recommendations of the Institute of Medicine (IOM) for GWG (38 % vs. 60 %, *p* < 0.05), and participants in the intervention group gained significantly less weight than those in the control group (14.1 vs. 15.6 kg, *p* < 0.05). Furthermore, the results suggested that weight retention was higher in the control group than in the intervention group at month four postpartum (pp) (3.3 vs. 2.1 kg, *p* = 0.09). There was no evidence of a difference in birth weight between the two groups.

The aim of the present study was to analyse the follow-up results of the FeLIPO trial regarding weight development of mothers and infants at 12 months pp. The main hypothesis was that a lifestyle intervention successfully limiting excessive GWG may also beneficially influence pp maternal and early childhood weight development. Next to the impact of lifestyle intervention during pregnancy on these parameters, associations with GWG were investigated. In addition, the potential association between dietary intake and physical activity during pregnancy with pp maternal and infant weight outcomes was studied.

## Methods

### Study design

The study design of the FeLIPO study has been described elsewhere [46]. In brief, it was a cluster-randomized controlled lifestyle intervention trial aiming to prevent excessive GWG. The study protocol was approved by the ethical committee of the Technische Universität München and registered in the German Clinical Trials Register (www.germanctr.de, DRKS00003801). The study population consisted of 250 healthy, pregnant women, who were recruited from eight gynaecological practices by their staff between February 2010 and August 2011 in the Munich area, Germany. Four practices were randomly assigned as intervention practices recruiting 167 pregnant women, whereas 83 women were recruited from the other four practices representing the control group. Women were recruited before the 18^th^ week of their pregnancy and gave their written, informed consent for participation. The FeLIPO intervention programme consisted of two individual counselling sessions given by trained researchers at the 20^th^ and 30^th^ week of gestation. Counselling sessions were structured and comprised three main components: a healthy diet, advice on physical activity and weight monitoring using the IOM charts of recommended GWG. Participants in the control group received routine prenatal care and an information leaflet with general statements about a healthy lifestyle during pregnancy.

### Measures and data collection

Pre-pregnancy weight, height, age and sociodemographic data were self-reported by the participants at the time of recruitment. At every antenatal visit, weight and pregnancy complications were routinely documented in the “Mutterpass” (maternity card). Practice staff copied maternity cards and birth records at the first postnatal visit. These records were used for data retrieval on birth data and complications during pregnancy and delivery. GWG was defined as the difference between body weight at the last obstetric visit prior to delivery (at a median time point of 38 weeks and 38.5 weeks in control and intervention group respectively, derived from maternity cards) and the self-reported pre-pregnancy weight.

Two follow-up interviews (phone call and/or e-mail) were arranged four months and one year pp in both groups. At these time points, self-reported data on maternal weights as well as breastfeeding data were collected. Weight retention at 12 months pp was defined as the difference in the self-reported weight at 12 months pp and the self-reported weight pre-pregnancy. In addition, women reported weight development of their children as assessed at the routine check-ups at the pediatricians, the so called “U-Untersuchungen”. These check-ups are conducted at defined time points and are documented in the “Kinder-Untersuchungsheft”, a small booklet, which is provided to all mothers in Germany. Body weight, height and health status at the U2 (3^rd^ to 10^th^ day pp), U3 (4^th^ to 6^th^ wk pp), U4 (3^rd^ to 4^th^ month pp), U5 (6^th^ to 7^th^ month pp) and U6 (10^th^ to 12^th^ month pp) were retrieved for the analysis.

Dietary intake was assessed using 7–day dietary records, which were completed three times (16^th^–18^th^ week [baseline], 26^th^–28^th^ week and 36^th^–38^th^ week of gestation) in the intervention and twice (16^th^–18^th^ week [baseline], 36^th^–38^th^ week of gestation) in the control group, respectively. Energy and macronutrient (fat, carbohydrate, protein and dietary fibre) intake was calculated using the nutrition software OptiDiet (version 5.0.0.029; Gesellschaft für optimierte Ernährung mbH-GOE, Linden, Germany). Dietary records with implausible energy intake were excluded from the statistical analysis. Underreporting of energy intake was defined using the cut off limit of Goldberg et al. (1.1 × BMR) [47]. BMR was calculated using the equation of Hronek et al. [48]. In the statistical analysis, we used the energy and macronutrient data at the 16^th^–18^th^ and 36^th^–38^th^ weeks of gestation together with the change between these measurements. Physical activity of women was assessed with the IPAQ’s long version in the 16^th^–18^th^ week, 26^th^–28^th^ week and 36^th^–38^th^ week of gestation in both groups [49].

### Statistical analyses

#### Maternal weight retention at 12 months pp

To assess differences in mean weight retention between the intervention and control group, a linear regression model was fitted with weight retention as the outcome variable and group as the fixed effect. In adjusted analyses, a random intercept for practice was added to the model, together with age, pre-pregnancy BMI and time of 12 month follow-up as fixed effects. The proportion of women with relevant weight retention (>5 kg) was compared between groups with an analogous logistic regression model. The threshold of 5 kg was chosen as it represents a clear increase in body weight associated with later obesity [50, 51]. Exclusive and total breastfeeding duration was compared between groups using t tests.

Linear regression models were fitted to the 12 month weight retention data to assess the association with GWG and the various components of dietary intake at 16 weeks, 36 weeks and the change over this time. Analyses were also performed adjusting for practice, age, pre-pregnancy BMI, time of follow-up and group. Associations between physical activity in the 16^th^–18^th^ week, 26^th^–28^th^ week and 36^th^–38^th^ week of pregnancy and 12 month weight retention were assessed in the same manner.

#### Infant weight outcomes

Mean differences in the weight of the infants between groups were assessed via separate linear regression models at each follow-up visit (U2, U3, U4, U5 and U6). The outcome variable was the infant’s weight with group as a fixed effect. In adjusted analyses, a random intercept for practice was added to the model together with fixed effects for maternal age, pre-pregnancy maternal BMI, infant age at follow-up, birth weight and group.

The association between GWG, dietary outcomes and physical activity during pregnancy with infant weight at U6 were assessed using linear regression analyses, in a similar manner to maternal weight retention.

All of the analyses are exploratory and the resulting p values have not been corrected for multiple testing. The results presented correspond to complete-case analyses. However, due to the presence of missing data (particularly for the dietary outcomes), we additionally conducted multiple imputation analyses. Results for the multiple imputation analyses are given as supplementary material (see Additional file 1: Tables S1–S3).

## Results

### Participant flow

**Fig. 1:**
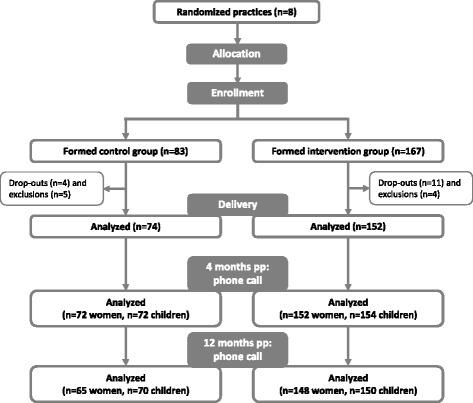
Study scheme of the FeLIPO study with follow-up

The participant flow in the FeLIPO study including the follow-up is shown in Fig. 1. Eighty three and 167 women were enrolled in the standard care and intervention group, respectively. From the second follow-up interview at 12 months pp, weight data were available for 213 women (65 in the standard care group and 148 in the intervention group). We were therefore able to follow-up 85.2 % of the participants initially recruited. Weight data from the U6 visit (10 to 12 months pp) were available for 220 infants (70 in the standard care and 150 in the intervention group). In some cases follow-up data were only available for children and not for their mothers as they either did not know their current body weight or did not want to report it. Eight women who withdrew from the study due to a miscarriage (2 women), abortion (2 women) or pregnancy complications (4 women) were excluded from the multiple imputation analyses.

### Maternal weight retention

**Fig. 2:**
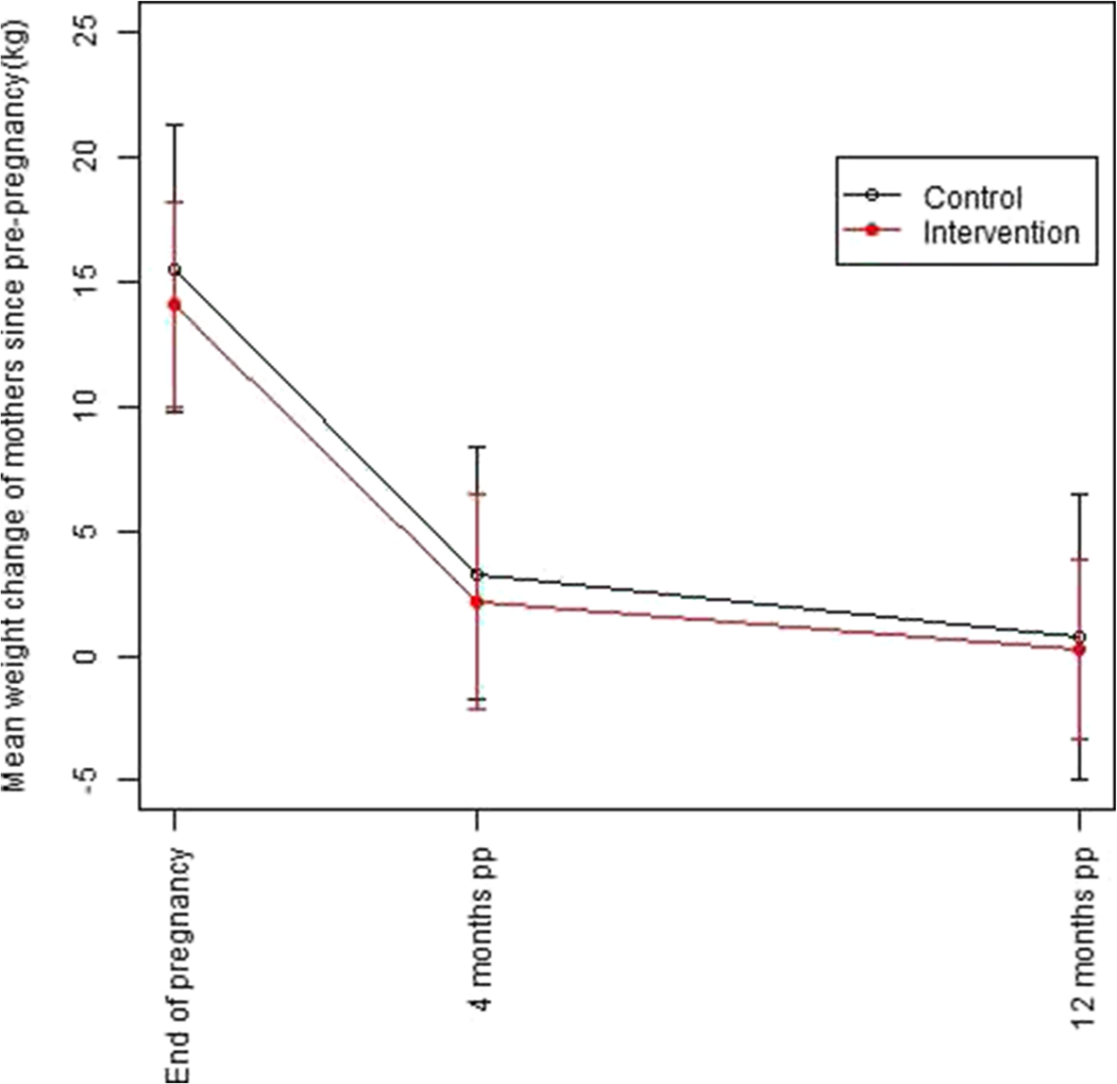
Mean weight change of the women in relation to pre-pregnancy weight. Body weight change (± s.d.) between pre-pregnancy and the last obstetric visit prior to delivery and body weight change until 4 and 12 months postpartum are displayed. The number of women providing data in the control group was 83, 74, 72 and 65 pre-pregnancy and at the mentioned time points respectively, and 167, 152, 152 and 148 in the intervention group

The mean weight changes for the women based on their pre-pregnancy weight according to group are shown in Fig. 2. At 12 months pp, the mean weight retention was higher in the control group (0.8 ± 5.7 kg) than in the intervention group (0.2 ± 3.6 kg). However, this difference in means was not statistically significant in unadjusted analysis (−0.5 kg (95 % CI: −1.8, 0.7), *p* = 0.402) or after adjusting for practice, age, pre-pregnancy BMI and time of 12 month follow-up (−1.0 kg (95 % CI: -3.2, 1.2), *p* = 0.321). Exclusive and total breastfeeding duration did not significantly differ between intervention and control group (130.7 vs. 116.3 days, *p* = 0.180; 232.1 vs. 219.4 days, *p* = 0.465). Further adjustment for breastfeeding duration did not change results. In the lifestyle intervention group, the proportion of women with weight retention >5 kg was 8.1 % 12 months pp whereas in the control group, 16.9 % retained >5 kg (OR: 0.43 (95 % CI: 0.18, 1.05), *p* = 0.061). This difference was statistically significant after adjusting for age, pre-pregnancy BMI, time of 12 month follow-up and practice (OR: 0.40 (95 % CI: 0.16, 0.97), *p* = 0.044).

For the whole study cohort, there is evidence of an association between greater GWG and increased 12 month weight retention. The unadjusted estimate for the effect of a 1 kg increase in GWG on 12 month weight retention was 0.4 kg (95 % CI: 0.3, 0.5), *p* < 0.001 and 0.4 kg (95 % CI: 0.2, 0.5), *p* < 0.001 after adjusting for potential confounders.

The results in Table 1 show that there is no evidence of an association between recorded dietary intake during pregnancy, in terms of energy and macronutrient intake, and weight retention at 12 months pp. Also for macronutrient intake calculated in percent of energy and physical activity data during pregnancy, no significant associations were found (data not shown).

**Table 1:** The association between reported dietary intake during pregnancy and weight retention at 12 months postpartum

Dietary component	Unadjusted effect size (95 % CI)	P^a^	Adjusted effect size (95 % CI)	P^b^
Energy intake (100 kcal/day)				
16^th^-18^th^ week	0.10 (−0.05, 0.26)	0.180	0.09 (−0.07, 0.24)	0.283
36^th^-38^th^ week	0.05 (−0.12, 0.23)	0.564	0.02 (−0.16, 0.20)	0.818
Change	0.06 (−0.11, 0.23)	0.499	0.06 (−0.12, 0.24)	0.495
Fat (g/day)				
16^th^-18^th^ week	0.02 (−0.01, 0.05)	0.306	0.01 (−0.02, 0.04)	0.461
36^th^-38^th^ week	0.01 (−0.02, 0.04)	0.598	0.00 (−0.03, 0.04)	0.844
Change	0.00 (−0.03, 0.04)	0.797	0.00 (−0.03, 0.04)	0.861
Carbohydrates (g/day)				
16^th^-18^th^ week	0.01 (−0.00, 0.02)	0.254	0.01 (−0.01, 0.02)	0.340
36^th^-38^th^ week	0.00 (−0.01, 0.01)	0.918	−0.00 (−0.01, 0.01)	0.744
Change	0.00 (−0.01, 0.02)	0.542	0.00 (−0.01, 0.02)	0.577
Protein (g/day)				
16^th^-18^th^ week	0.03 (−0.00, 0.07)	0.086	0.03 (−0.01, 0.07)	0.129
36^th^-38^th^ week	0.04 (−0.01, 0.08)	0.111	0.04 (−0.00, 0.08)	0.081
Change	0.02 (−0.02, 0.06)	0.372	0.03 (−0.02, 0.07)	0.212
Fibre (g/day)				
16^th^-18^th^ week	0.01 (−0.08, 0.10)	0.777	−0.01 (−0.10, 0.09)	0.881
36^th^-38^th^ week	−0.02 (−0.11, 0.08)	0.688	−0.04 (−0.13, 0.06)	0.461
Change	0.02 (−0.09, 0.13)	0.695	0.01 (−0.10, 0.13)	0.820

Data from 233 women, 170 women and 168 women were available for the 16^th^-18^th^ week, 36^th^-38^th^ week and dietary change analyses respectively

Effect sizes from regression models as estimated marginal mean difference (95 % CI)

^a^unadjusted model

^b^linear mixed model adjusted for practice (random factor), maternal age, pre-pregnancy BMI, time of follow-up and group

### Infant weight progression

**Fig. 3:**
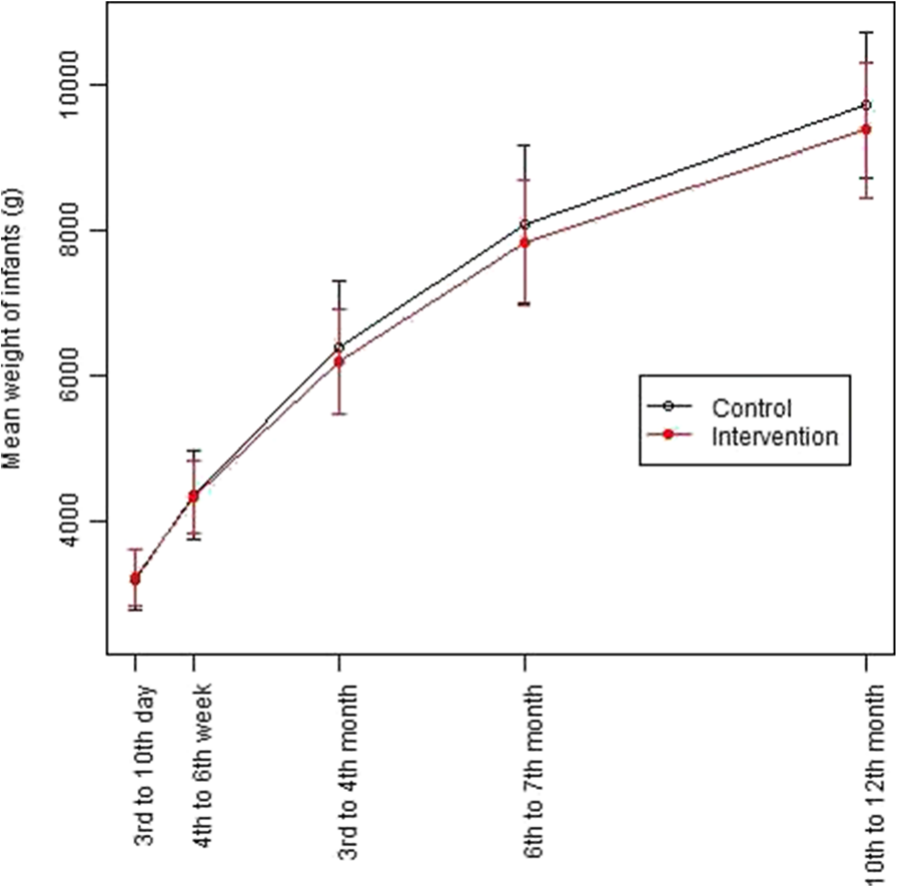
Mean weight of the infants (± s.d.) at each time point according to group

The weight trajectories for the infants according to the group to which their mother was assigned are given in Fig. 3. The corresponding results for between group differences at U2, U3, U4, U5 and U6 are given in Table 2. There is no evidence of a difference in weight trajectories up to and including U5 (6^th^ to 7^th^ month pp). At U6 (10^th^ to 12^th^ month pp), there is some evidence that infants born to mothers in the intervention group weighed less than control group infants (−354 g (95 % CI: −626, −82), *p* = 0.011). However, the estimated mean difference between the groups at 12 months was not statistically significant after adjusting for practice, maternal age, pre-pregnancy maternal BMI, infant age at follow-up and birth weight (−257 g (95 % CI: −578, 65), *p* = 0.099). After additional adjustment for breastfeeding duration, body weights were also not statistically significantly different between the two groups (-220 g (95 % CI: -538, 98), *p* = 0.141).

**Table 2:** Infant weight at follow-up visits and the estimated mean weight differences between the groups

Age		Control group	Intervention group	Unadjusted effect size (95 % CI)	P^a^	Adjusted effect size (95%CI)	P^b^
3^rd^ to 10^th^ day	n	72	151				
	Time after birth (days)	3 (3–4)	3 (2–3.25)				
	Weight (g)	3200 ± 414	3227 ± 392	27 (−86, 139)	0.640	21 (−23, 65)	0.283
4^th^ to 6^th^ week	n	73	153				
	Time after birth (days)	33 (29–37)	32 (28–37)				
	Weight (g)	4362 ± 607	4346 ± 499	−16 (−166, 134)	0.832	−29 (−150, 93)	0.584
3^rd^ to 4^th^ month	n	72	154				
	Time after birth (days)	103 (93–113)	103 (93–114)				
	Weight (g)	6395 ± 920	6209 ± 725	−186 (−409, 37)	0.102	−191 (−466, 84)	0.140
6^th^ to 7^th^ month	n	70	151				
	Time after birth (days)	191 (182–206)	195 (182–208)				
	Weight (g)	8079 ± 1085	7845 ± 859	−234 (−501, 33)	0.085	−209 (−511, 93)	0.142
10^th^ to 12^th^ month	n	70	150				
	Time after birth (days)	365 (350–377)	358 (342–371)				
	Weight (g)	9736 ± 999	9382 ± 931	−354 (−626, −82)	0.011	−257 (−578, 65)	0.099

Data are given as means ± SD or median (interquartile range)

Effect sizes from regression models as estimated marginal mean difference (95 % CI)

^a^ unadjusted model

^b^ linear mixed model adjusted for practice (random factor), maternal age and pre-pregnancy BMI, infant age at follow-up and birth weight

There is some evidence of an association between greater GWG and increased weight of infants at 12 months of age. In unadjusted analysis, the estimated effect of a 1 kg increase in GWG is 39 g (95 % CI: 10, 68), *p* = 0.009. After adjusting for confounders, the estimated effect size is smaller and not statistically significant: 26 g (95 % CI: −2, 54), *p* = 0.074.

The results in Table 3 show that there is no evidence of an association between recorded dietary intake during pregnancy and infant’s weight at 12 months. Also for macronutrient intake calculated in percent of energy and physical activity data during pregnancy, no significant associations were found (data not shown).

**Table 3:** The association between reported dietary intake during pregnancy and infant weight at 12 months

Dietary component	Unadjusted effect size (95 % CI)	P^a^	Adjusted effect size (95 % CI)	P^b^
Energy intake (100 kcal/day)				
16^th^-18^th^ week	−17 (−52, 17)	0.317	−9 (41, 23)	0.575
36^th^-38^th^ week	13 (−26, 53)	0.510	16 (−22, 54)	0.402
Change	5 (−35, 44)	0.813	4 (−34, 41)	0.853
Fat (g/day)				
16^th^-18^th^ week	−3 (−10, 4)	0.359	−2 (−8, 4)	0.537
36^th^-38^th^ week	2 (−6, 9)	0.650	2 (−6, 9)	0.659
Change	2 (−6, 9)	0.652	1 (−6, 8)	0.803
Carbohydrates (g/day)				
16^th^-18^th^ week	−1 (−4, 1)	0.341	−1 (−3, 2)	0.618
36^th^-38^th^ week	1 (−2, 4)	0.514	1 (−1, 4)	0.355
Change	0 (−3, 3)	0.879	0 (−2, 3)	0.829
Protein (g/day)				
16^th^-18^th^ week	−1 (−9, 8)	0.896	1 (−7, 9)	0.794
36^th^-38^th^ week	2 (−8, 12)	0.672	2 (−7, 11)	0.677
Change	−2 (−12, 8)	0.693	−3 (−12, 6)	0.547
Fibre (g/day)				
16^th^-18^th^ week	−7 (28, 13)	0.486	4 (−16, 23)	0.723
36^th^-38^th^ week	−1 (−22, 20)	0.927	2 (−18, 23)	0.828
Change	−3 (−29, 23)	0.815	−8 (33, 16)	0.500

Effect sizes from regression models as estimated marginal mean difference (95 % CI)

^a^ unadjusted model

^b^ linear mixed model adjusted for practice (random factor), maternal age and pre-pregnancy BMI, infant age at follow-up, birth weight and group

Multiple imputation results (available as supplementary material, see Additional file 1) are consistent with the complete-case results presented.

## Discussion

We investigated mothers’ and infants’ 12-month pp follow-up weight data after lifestyle intervention during pregnancy. The intervention had resulted in reduced mean GWG in pregnant women. 12 months after delivery, the amount of weight retained did not significantly differ between the former intervention and control groups. A tendency towards lower mean weight retention among women who had received the intervention was nevertheless visible. The proportion of women with relevant weight retention (>5 kg) 12 months pp was by trend higher in the group not receiving lifestyle intervention during pregnancy. At 6 months pp, a tendency towards lower weight retention was also found in other intervention studies [43, 52, 53]. Longer-term pp weight data (>6 months) after lifestyle intervention in pregnancy is scarce and controversial. Behavioural intervention was found to be associated with reduced weight retention 12 months pp in an American study [54]. In obese women, a difference in weight change between early pregnancy and 12 months after childbirth due to a lifestyle intervention was indicated by Claesson et al. [52]. By contrast, weight retention was reduced by antenatal lifestyle intervention only in the subgroup of low-income overweight women in a study of Olson et al. [55], and Althuizen et al. [56] could not find any effect on weight retention. As evidence for long-term effects of lifestyle interventions on pp weight development of women is limited, further research in large intervention studies with adequate follow-up duration is required.

An association between GWG and pp weight retention is well established from cohort studies. For the whole cohort of the present study, higher weight gain during pregnancy was associated with increased weight retention 12 months pp (0.4 kg weight retention per 1 kg increase in GWG). High GWG has been linked to increased short-term weight retention in several observational studies [20, 57–59]. Nehring et al. [18] concluded in a meta-analysis that GWG is also associated with long-term weight retention, being in line with the results of a recently published large prospective cohort study [60]. However, GWG is not the only factor influencing weight retention after gestation. Other factors discussed in this context are breastfeeding [22, 61], the women’s educational level [61], parity [62], diet [63] and physical activity [22, 63]. In conclusion, the extent of GWG seems to be not the only but an important influencing factor for long-term pp weight retention. Having an effect on weight retention after pregnancy, high GWG has also been linked to women’s long-term BMI development and obesity risk [17, 21–23, 61, 64–67]. Lifestyle counselling has the potential to favour adequate GWG and could by this means also be of help in the prevention of long-term development of overweight and obesity in women.

In the present study, counselling lessons were given only during pregnancy. Prolonging the intervention to the pp period may reinforce the reducing effect on maternal weight retention. However, Wilkinson *et al.* [68] recently found no impact of a pp intervention programme on pp body weight, whereas Huang *et al.* [53] showed that starting intervention during pregnancy and continuing it pp can lead to reduced weight retention. Additional counselling lessons after childbirth subsequent to lifestyle intervention during pregnancy may thus be beneficial, but need to be evaluated in more clinical studies.

Weight data of children were collected for U2 (3^rd^ to 10^th^ day pp) to U6 (10^th^ to 12^th^ month pp). At U6, infants born to mothers in the intervention group tended to weigh less than control group infants. This may partly be explained by a beneficial effect of lifestyle counselling during pregnancy on the offspring’s weight development. Next to intrauterine mechanisms during gestation potentially related to a healthier lifestyle of women in the intervention group, other triggers could be responsible for this observation. For example, breastfeeding and its duration [69–73] seems to be of relevance for the weight development of children. Lifestyle counselling was not continued after delivery and topics such as nutrition of the infant were not covered in the lessons. It can nevertheless not be completely ruled out that women encouraged towards a healthy lifestyle during pregnancy developed increased consciousness to healthy feeding their children. Following adjustment for breastfeeding, the estimated weight difference between the groups was reduced. Although mothers in the intervention group tended to breastfeed their offspring a bit longer, no statistically significant group difference could be found.

Independently of the allocation to the intervention or control group, there was some evidence for an association between higher GWG and increased infant body weight at the age of 10–12 months. An association between high GWG and increased weight of children has also been reported in observational studies [74, 75]. This speaks in favour of intrauterine effects during pregnancy on later weight development of children. Nevertheless, the genetic background as well as the environment shared by mother and infant are factors that should not be neglected in this context [76, 77]. Breastfeeding, for example, seems to mitigate the association between GWG and childhood anthropometrics [73]. As implied by the slight weight difference only visible at the 12 months measurement point in this study, differences in body weight may arise delayed in time. As also proposed by others [78], follow-up of children after lifestyle intervention in pregnancy is essential for the clarification of in utero effects during gestation on later overweight or obesity. Intervention studies targeting GWG with adequate follow-up periods are thus urgently required.

As published previously [46], lifestyle counselling during pregnancy could be shown to have an impact on the caloric intake of the pregnant women. In the context of this follow-up analysis, energy and macronutrient intake during pregnancy was analysed with respect to maternal weight retention (12 months pp) and weight development of their children. However, no association with weight retention or infant body weight at the age of 12 months could be found. It seems to be difficult to establish a relation between nutrient intake in pregnancy and maternal weight retention or weight development of their children. However, this may be also due to the fact that the pp situation is too complex as there are many factors which could influence this association. Potential associations between the intake of single nutrients and outcome parameters may be too weak to be clearly identifiable. Analysing dietary patterns instead of single nutrients could be a more promising approach in the identification of potential associations with the mentioned weight parameters.

A limitation of this study is the difference in baseline characteristics, e.g. pre-pregnancy weight, between intervention and control group. Despite including these parameters as adjustment variables, an impact of these differences on the results cannot be excluded. Additionally, more women participated in practices allocated to the intervention group than in control practices, causing unequal group sizes. Another limitation is the missing verifiability of maternal weight data. While body weights of children were documented by pediatricians as part of the routine health care, maternal pre-pregnancy and 12 month pp weight data were self-reported. However, self-reported maternal weight data have been reported to provide valid estimates [79] and are widely used in lifestyle intervention studies during pregnancy [80]. Additional controlling for paternal anthropometrics might also have an influence on infant weight data. Physical activity and nutrition behaviour of women after birth as well as food intake of children was not studied, which may have added valuable additional information.

To confirm the outcomes of the present pilot study, we initiated another large lifestyle intervention study (GeliS, acronym for “Gesund leben in der Schwangerschaft”/healthy living in pregnancy) [81]. This currently ongoing large-scale intervention study aims to recruit and follow-up 2500 pregnant women. With a study population tenfold as large as the one of the FeLIPO study spread over ten regions of Bavaria, we aim to overcome these limitations. An enhanced number of counselling sessions, reaching from early pregnancy to the pp period, will intensify the lifestyle intervention. In order to investigate long-term effects on weight trajectories of children, an extended follow-up period up to the age of 5 years including assessment of dietary behaviour of children is planned.

## Conclusion

In addition to reducing the amount of pregnancies with excessive weight gain, counselling on a healthy diet, physical activity and weight monitoring during pregnancy may have the potential to limit 1-year post-partum weight retention. Children of women receiving lifestyle counselling may be at lower risk for the long-term development of overweight, but both outcomes need to be further confirmed. Large-scale intervention trials such as the currently ongoing GeliS study are urgently required to clearly establish the potential benefits of a lifestyle intervention programme onto the health of pregnant women and their offspring.

## Additional file

Additional file 1: **Supplementary material, results from multiple imputation analysis.** (PDF 56 kb)

## References

[1] World Health Organization: WHO | Obesity and overweight: Fact Sheet N°311 [http://www.who.int/mediacentre/factsheets/fs311/en/index.html].

[2] Kurth B. Erste Ergebnisse aus der “Studie zur Gesundheit Erwachsener in Deutschland“ (DEGS). Bundesgesundheitsbl, 2012:980–990.

[3] Mensink GBM, Schienkiewitz A, Haftenberger M, Lampert T, Ziese T, Scheidt-Nave C (2013). Übergewicht und Adipositas in Deutschland: Ergebnisse der Studie zur Gesundheit Erwachsener in Deutschland (DEGS1). Bundesgesundhbl Gesundheitsforsch Gesundheitsschutz.

[4] Catalano PM, Ehrenberg HM (2006). The short- and long-term implications of maternal obesity on the mother and her offspring. BJOG.

[5] Guelinckx I, Devlieger R, Beckers K, Vansant G (2008). Maternal obesity: pregnancy complications, gestational weight gain and nutrition. Obes Rev.

[6] Davies GAL, Maxwell C, McLeod L, Gagnon R, Basso M, Bos H (2010). SOGC clinical practice guidelines: obesity in pregnancy. No. 239, February 2010. Int J Gynaecol Obstet.

[7] Nelson SM, Matthews P, Poston L (2010). Maternal metabolism and obesity: modifiable determinants of pregnancy outcome. Hum Reprod Update.

[8] Adamo KB, Ferraro ZM, Brett KE (2012). Can we modify the intrauterine environment to halt the intergenerational cycle of obesity?. Int J Environ Res Publ Health.

[9] Gaudet L, Ferraro ZM, Wen SW, Walker M (2014). Maternal obesity and occurrence of fetal macrosomia: a systematic review and meta-analysis. BioMed Res Int.

[10] Viswanathan M, Siega-Riz AM, Moos MK, Deierlein A, Mumford S, Knaack J, et al. Outcomes of maternal weight gain. Evid Rep Technol Assess. 2008;1–223. PMC4781425 18620471

[11] Hedderson MM, Gunderson EP, Ferrara A (2010). Gestational weight gain and risk of gestational diabetes mellitus. Obstet Gynecol.

[12] Margerison-Zilko CE, Rehkopf D, Abrams B (2010). Association of maternal gestational weight gain with short- and long-term maternal and child health outcomes. Am J Obstet Gynecol.

[13] Durie DE, Thornburg LL, Glantz JC (2011). Effect of second-trimester and third-trimester rate of gestational weight gain on maternal and neonatal outcomes. Obstet Gynecol.

[14] Jang DG, Jo YS, Lee GS (2011). Effect of pre-pregnancy body mass index and weight gain during pregnancy on the risk of emergency cesarean section in nullipara. Arch Gynecol Obstet.

[15] Gibson KS, Waters TP, Catalano PM (2012). Maternal weight gain in women who develop gestational diabetes mellitus. Obstet Gynecol.

[16] Liu Y, Dai W, Dai X, Li Z (2012). Prepregnancy body mass index and gestational weight gain with the outcome of pregnancy: a 13-year study of 292,568 cases in China. Arch Gynecol Obstet.

[17] Linné Y, Dye L, Barkeling B, Rössner S (2004). Long-term weight development in women: a 15-year follow-up of the effects of pregnancy. Obes Res.

[18] Nehring I, Schmoll S, Beyerlein A, Hauner H, Von Kries R (2011). Gestational weight gain and long-term postpartum weight retention: a meta-analysis. Am J Clin Nutr.

[19] Begum F, Colman I, McCargar LJ, Bell RC (2012). Gestational weight gain and early postpartum weight retention in a prospective cohort of Alberta women. J Obstet Gynaecol Can.

[20] Rode L, Kjærgaard H, Ottesen B, Damm P, Hegaard HK (2012). Association between gestational weight gain according to body mass index and postpartum weight in a large cohort of Danish women. Matern Child Health J.

[21] Gunderson EP, Abrams B, Selvin S (2000). The relative importance of gestational gain and maternal characteristics associated with the risk of becoming overweight after pregnancy. Int J Obes Relat Metab Disord.

[22] Rooney BL, Schauberger CW (2002). Excess pregnancy weight gain and long-term obesity: one decade later. Obstet Gynecol.

[23] Mamun AA, Kinarivala M, O’Callaghan MJ, Williams GM, Najman JM, Callaway LK (2010). Associations of excess weight gain during pregnancy with long-term maternal overweight and obesity: evidence from 21 y postpartum follow-up. Am J Clin Nutr.

[24] DeVader SR, Neeley HL, Myles TD, Leet TL (2007). Evaluation of gestational weight gain guidelines for women with normal prepregnancy body mass index. Obstet Gynecol.

[25] Ferraro ZM, Barrowman N, Prud’homme D, Walker M, Wen SW, Rodger M (2012). Excessive gestational weight gain predicts large for gestational age neonates independent of maternal body mass index. J Matern Fetal Neonatal Med.

[26] Lee JM, Kim MJ, Kim MY, Han JY, Ahn HK, Choi JS (2014). Gestational weight gain is an important risk factor for excessive fetal growth. Obstet Gynecol Sci.

[27] Moreira P, Padez C, Mourão-Carvalhal I, Rosado V (2007). Maternal weight gain during pregnancy and overweight in Portuguese children. Int J Obes (Lond).

[28] Oken E, Taveras EM, Kleinman KP, Rich-Edwards JW, Gillman MW. Gestational weight gain and child adiposity at age 3 years. Am J Obstet Gynecol 2007, 196:322.e1-8. 10.1016/j.ajog.2006.11.027 PMC1899090 17403405

[29] Wrotniak BH, Shults J, Butts S, Stettler N (2008). Gestational weight gain and risk of overweight in the offspring at age 7 y in a multicenter, multiethnic cohort study. Am J Clin Nutr.

[30] Olson CM, Strawderman MS, Dennison BA (2009). Maternal weight gain during pregnancy and child weight at age 3 years. Matern Child Health J.

[31] von Kries R, Ensenauer R, Beyerlein A, Amann-Gassner U, Hauner H, Rosario AS (2011). Gestational weight gain and overweight in children: Results from the cross-sectional German KiGGS study. Int J Pediatr Obes.

[32] Beyerlein A, Nehring I, Rzehak P, Heinrich J, Müller MJ, Plachta-Danielzik S (2012). Gestational weight gain and body mass index in children: results from three german cohort studies. PloS One.

[33] Margerison-Zilko CE, Shrimali BP, Eskenazi B, Lahiff M, Lindquist AR, Abrams BF (2012). Trimester of maternal gestational weight gain and offspring body weight at birth and age five. Matern Child Health J.

[34] Dello Russo M, Ahrens W, De Vriendt T, Marild S, Molnar D, Moreno LA (2013). Gestational weight gain and adiposity, fat distribution, metabolic profile, and blood pressure in offspring: the IDEFICS project. Int J Obes Relat Metab Disord.

[35] Ensenauer R, Chmitorz A, Riedel C, Fenske N, Hauner H, Nennstiel-Ratzel U (2013). Effects of suboptimal or excessive gestational weight gain on childhood overweight and abdominal adiposity: results from a retrospective cohort study. Int J Obes (Lond).

[36] Oken E, Rifas-Shiman SL, Field AE, Frazier AL, Gillman MW (2008). Maternal gestational weight gain and offspring weight in adolescence. Obstet Gynecol.

[37] Mamun AA, O’Callaghan M, Callaway L, Williams G, Najman J, Lawlor DA (2009). Associations of gestational weight gain with offspring body mass index and blood pressure at 21 years of age: evidence from a birth cohort study. Circulation.

[38] Reynolds RM, Osmond C, Phillips DIW, Godfrey KM (2010). Maternal BMI, parity, and pregnancy weight gain: influences on offspring adiposity in young adulthood. J Clin Endocrinol Metab.

[39] Schack-Nielsen L, Michaelsen KF, Gamborg M, Mortensen EL, Sørensen TIA (2010). Gestational weight gain in relation to offspring body mass index and obesity from infancy through adulthood. Int J Obes (Lond).

[40] Laitinen J, Jääskeläinen A, Hartikainen A, Sovio U, Vääräsmäki M, Pouta A (2012). Maternal weight gain during the first half of pregnancy and offspring obesity at 16 years: a prospective cohort study. BJOG.

[41] Wolff S, Legarth J, Vangsgaard K, Toubro S, Astrup A (2008). A randomized trial of the effects of dietary counseling on gestational weight gain and glucose metabolism in obese pregnant women. Int J Obes (Lond).

[42] Streuling I, Beyerlein A, von Kries R (2010). Can gestational weight gain be modified by increasing physical activity and diet counseling? A meta-analysis of interventional trials. Am J Clin Nutr.

[43] Phelan S, Phipps MG, Abrams B, Darroch F, Schaffner A, Wing RR (2011). Randomized trial of a behavioral intervention to prevent excessive gestational weight gain: the Fit for Delivery Study. Am J Clin Nutr.

[44] Vinter CA, Jensen DM, Ovesen P, Beck-Nielsen H, Jørgensen JS (2011). The LiP (Lifestyle in Pregnancy) study: a randomized controlled trial of lifestyle intervention in 360 obese pregnant women. Diabetes Care.

[45] Ronnberg A, Ostlund I, Fadl H, Gottvall T, Nilsson K. Intervention during pregnancy to reduce excessive gestational weight gain-a randomised controlled trial. BJOG. 2014. 10.1111/1471-0528.13131 25367823

[46] Rauh K, Gabriel E, Kerschbaum E, Schuster T, von Kries R, Amann-Gassner U (2013). Safety and efficacy of a lifestyle intervention for pregnant women to prevent excessive maternal weight gain: a cluster-randomized controlled trial. BMC Pregnancy Childbirth.

[47] Goldberg GR, Black AE, Jebb SA, Cole TJ, Murgatroyd PR, Coward WA (1991). Critical evaluation of energy intake data using fundamental principles of energy physiology: 1. Derivation of cut-off limits to identify under-recording. Eur J Clin Nutr.

[48] Hronek M, Zadak Z, Hrnciarikova D, Hyspler R, Ticha A (2009). New equation for the prediction of resting energy expenditure during pregnancy. Nutrition.

[49] Craig CL, Marshall AL, Sjöström M, Bauman AE, Booth ML, Ainsworth BE (2003). International physical activity questionnaire: 12-country reliability and validity. Med Sci Sports Exerc.

[50] Gunderson EP, Abrams B (1999). Epidemiology of gestational weight gain and body weight changes after pregnancy. Epidemiol Rev.

[51] Ohlin A, Rössner S (1990). Maternal body weight development after pregnancy. Int J Obes.

[52] Claesson I, Sydsjö G, Brynhildsen J, Blomberg M, Jeppsson A, Sydsjö A (2011). Weight after childbirth: a 2-year follow-up of obese women in a weight-gain restriction program. Acta Obstet Gynecol Scand.

[53] Huang T, Yeh C, Tsai Y (2011). A diet and physical activity intervention for preventing weight retention among Taiwanese childbearing women: a randomised controlled trial. Midwifery.

[54] Phelan S, Phipps MG, Abrams B, Darroch F, Grantham K, Schaffner A (2014). Does behavioral intervention in pregnancy reduce postpartum weight retention? Twelve-month outcomes of the Fit for Delivery randomized trial. Am J Clin Nutr.

[55] Olson CM, Strawderman MS, Reed RG (2004). Efficacy of an intervention to prevent excessive gestational weight gain. Am J Obstet Gynecol.

[56] Althuizen E, van der Wijden CL, van Mechelen W, Seidell JC, van Poppel MNM (2013). The effect of a counselling intervention on weight changes during and after pregnancy: a randomised trial. BJOG.

[57] Butte NF, Ellis KJ, Wong WW, Hopkinson JM, Smith EO (2003). Composition of gestational weight gain impacts maternal fat retention and infant birth weight. Am J Obstet Gynecol.

[58] Nohr EA, Vaeth M, Baker JL, Sørensen TI, Olsen J, Rasmussen KM (2008). Combined associations of prepregnancy body mass index and gestational weight gain with the outcome of pregnancy. Am J Clin Nutr.

[59] Huang T, Wang H, Dai F (2010). Effect of pre-pregnancy body size on postpartum weight retention. Midwifery.

[60] Haugen M, Brantsæter AL, Winkvist A, Lissner L, Alexander J, Oftedal B (2014). Associations of pre-pregnancy body mass index and gestational weight gain with pregnancy outcome and postpartum weight retention: a prospective observational cohort study. BMC Pregnancy Childbirth.

[61] Amorim AR, Rössner S, Neovius M, Lourenço PM, Linné Y (2007). Does excess pregnancy weight gain constitute a major risk for increasing long-term BMI?. Obesity (Silver Spring).

[62] Maddah M, Nikooyeh B (2009). Weight retention from early pregnancy to three years postpartum: a study in Iranian women. Midwifery.

[63] Ng S, Cameron CM, Hills AP, McClure RJ, Scuffham PA (2014). Socioeconomic disparities in prepregnancy BMI and impact on maternal and neonatal outcomes and postpartum weight retention: the EFHL longitudinal birth cohort study. BMC Pregnancy Childbirth.

[64] Gore SA, Della Brown M, West DS (2003). The role of postpartum weight retention in obesity among women: a review of the evidence. Ann Behav Med.

[65] Fraser A, Tilling K, Macdonald-Wallis C, Hughes R, Sattar N, Nelson SM (2011). Associations of gestational weight gain with maternal body mass index, waist circumference, and blood pressure measured 16 y after pregnancy: the Avon Longitudinal Study of Parents and Children (ALSPAC). Am J Clin Nutr.

[66] Mannan M, Doi, Suhail AR, Mamun AA (2013). Association between weight gain during pregnancy and postpartum weight retention and obesity: a bias-adjusted meta-analysis. Nutr Rev.

[67] McClure CK, Catov JM, Ness R, Bodnar LM (2013). Associations between gestational weight gain and BMI, abdominal adiposity, and traditional measures of cardiometabolic risk in mothers 8 y postpartum. Am J Clin Nutr.

[68] Wilkinson SA, van der Pligt P, Gibbons KS, McIntyre HD (2015). Trial for Reducing Weight Retention in New Mums: a randomised controlled trial evaluating a low intensity, postpartum weight management programme. J Hum Nutr Diet.

[69] Arenz S, Rückerl R, Koletzko B, Von Kries R (2004). Breast-feeding and childhood obesity--a systematic review. Int J Obes Relat Metab Disord.

[70] Harder T, Bergmann R, Kallischnigg G, Plagemann A (2005). Duration of breastfeeding and risk of overweight: a meta-analysis. Am J Epidemiol.

[71] Owen CG, Martin RM, Whincup PH, Smith GD, Cook DG (2005). Effect of infant feeding on the risk of obesity across the life course: a quantitative review of published evidence. Pediatrics.

[72] Beyerlein A, Von Kries R (2011). Breastfeeding and body composition in children: will there ever be conclusive empirical evidence for a protective effect against overweight?. Am J Clin Nutr.

[73] Zhu Y, Hernandez LM, Dong Y, Himes JH, Hirschfeld S, Forman MR (2015). Longer breastfeeding duration reduces the positive relationships among gestational weight gain, birth weight and childhood anthropometrics. J Epidemiol Community Health.

[74] Nehring I, Lehmann S, von Kries R (2013). Gestational weight gain in accordance to the IOM/NRC criteria and the risk for childhood overweight: a meta-analysis. Pediatr Obes.

[75] Mamun AA, Mannan M, Doi SAR (2014). Gestational weight gain in relation to offspring obesity over the life course: a systematic review and bias-adjusted meta-analysis. Obes Rev.

[76] Ebbeling CB, Pawlak DB, Ludwig DS (2002). Childhood obesity: public-health crisis, common sense cure. Lancet.

[77] Lawlor DA, Lichtenstein P, Fraser A, Långström N (2011). Does maternal weight gain in pregnancy have long-term effects on offspring adiposity? A sibling study in a prospective cohort of 146,894 men from 136,050 families. Am J Clin Nutr.

[78] Dodd JM, Grivell RM, Crowther CA, Robinson JS (2010). Antenatal interventions for overweight or obese pregnant women: a systematic review of randomised trials. BJOG.

[79] Lederman SA, Paxton A (1998). Maternal reporting of prepregnancy weight and birth outcome: consistency and completeness compared with the clinical record. Matern Child Health J.

[80] Tanentsapf I, Heitmann BL, Adegboye ARA (2011). Systematic review of clinical trials on dietary interventions to prevent excessive weight gain during pregnancy among normal weight, overweight and obese women. BMC Pregnancy Childbirth.

[81] Rauh K, Kunath J, Rosenfeld E, Kick L, Ulm K, Hauner H (2014). Healthy living in pregnancy: a cluster-randomized controlled trial to prevent excessive gestational weight gain - rationale and design of the GeliS study. BMC Pregnancy Childbirth.

