# Supplementary material for: Lifestyle intervention to prevent excessive maternal weight gain: mother and infant follow-up at 12 months postpartum
Source: BMC Pregnancy Childbirth. 2015 Oct 15;15:265. doi: 10.1186/s12884-015-0701-2 (PMC4608142; doi:10.1186/s12884-015-0701-2)
Supplement: Supplementary file 1 — Supplementary material, results from multiple imputation analysis. (PDF 56 kb) [file 12884_2015_701_MOESM1_ESM.pdf]

Supplement

Maternal weight retention

Following multiple imputation, the estimated mean difference in 12 month weight retention between groups is -0.4 kg (-1.7, 0.9), p = 0.573 in unadjusted analysis and -0.4 kg (-1.9, 1.1), p = 0.604 in adjusted analysis. The estimated effect of GWG on 12 month weight retention is 0.3 kg (0.1, 0.4), $p < 0.001$ in both unadjusted and adjusted analyses. The results for the association between dietary intake during pregnancy and weight retention at 12 months post-partum are given in Table 1.

Infant outcomes

The estimated between group differences in infant weight at each follow-up time point, following multiple imputation, are given in Table 2. The effect of a 1 kg increase in GWG on the weight of infants at 12 months is estimated as 30 g (0, 61), p = 0.052 in unadjusted analysis, and 21 g (-8, 50), p = 0.150 in adjusted analysis. The results for the association between dietary intake during pregnancy and weight of infants at 12 months are given in Table 3.

Table 1. The association between reported dietary intake during pregnancy and weight retention at 12 months post-partum (following multiple imputation)

Dietary component	Unadjusted effect size (95% CI)	P[a]	Adjusted effect size (95% CI)	P[b]
**Energy intake (100kcal/day)**				
**16th-18th week**	0.10 (-0.06, 0.26)	0.233	0.08 (-0.08, 0.24)	0.330
**36th-38th week**	0.07 (-0.10, 0.25)	0.405	0.05 (-0.12, 0.23)	0.545
**Change**	-0.03 (-0.21, 0.14)	0.696	-0.03 (-0.21, 0.14)	0.720
**Fat (g/day)**				
**16th-18th week**	0.02 (-0.02, 0.05)	0.336	0.01 (-0.02, 0.04)	0.478
**36th-38th week**	0.01 (-0.02, 0.04)	0.564	0.01 (-0.02, 0.04)	0.676
**Change**	-0.01 (-0.04, 0.03)	0.701	-0.00 (-0.04, 0.03)	0.793
**Carbohydrates (g/day)**				
**16th-18th week**	0.01 (-0.01, 0.02)	0.316	0.01 (-0.01, 0.02)	0.382
**36th-38th week**	0.00 (-0.01, 0.02)	0.633	0.00 (-0.01, 0.01)	0.818
**Change**	-0.00 (-0.02, 0.01)	0.661	-0.00 (-0.02, 0.01)	0.577
**Protein (g/day)**				
**16th-18th week**	0.03 (-0.01,0.07)	0.141	0.03 (-0.01, 0.06)	0.206
**36th-38th week**	0.02 (-0.02, 0.06)	0.306	0.02 (-0.02, 0.06)	0.295
**Change**	-0.01 (-0.05, 0.04)	0.817	-0.00 (-0.04, 0.04)	0.967
**Fibre (g/day)**				
**16th-18th week**	0.01 (-0.09, 0.10)	0.861	-0.02 (-0.12, 0.08)	0.705
**36th-38th week**	-0.00 (-0.10, 0.10)	0.949	-0.02 (-0.12, 0.08)	0.654
**Change**	-0.01 (-0.11, 0.10)	0.882	-0.01 (-0.12, 0.10)	0.907

Effect sizes from regression models as estimated marginal mean difference (95% CI)
a) unadjusted model
b) linear mixed model adjusted for practice (random factor), maternal age, pre-pregnancy BMI, time of follow-up and group

Table 2. Infant weight at follow-up visits in each group and the estimated mean weight differences between the groups (following multiple imputation)

Time point	Unadjusted effect size (95% CI)	P [a)]	Adjusted effect size (95%CI)	P [b)]
**U2**	8 (-101, 116)	0.892	25 (-15, 64)	0.220
**U3**	-22 (-172, 129)	0.777	-18 (-115, 78)	0.707
**U4**	-176 (-394, 43)	0.114	-166 (-381, 49)	0.130
**U5**	-250 (-514,14)	0.063	-203 (-446, 40)	0.101
**U6**	-368 (-634, -103)	0.007	-259 (-516,-2)	0.048

Effect sizes from regression models as estimated marginal mean difference (95% CI)
a) unadjusted model
b) linear mixed model adjusted for practice (random factor), maternal age and pre-pregnancy BMI, infant age at follow-up and birth weight

Table 3. The association between reported dietary intake during pregnancy and infant weight at 12 months (following multiple imputation)

Dietary component	Unadjusted effect size (95% CI)	P[a]	Adjusted effect size (95% CI)	P[b]
**Energy intake (100kcal/day)**				
**16th-18th week**	-18 (-51, 15)	0.278	-10 (-41, 21)	0.524
**36th-38th week**	1 (-41, 42)	0.977	6 (-31, 43)	0.756
**Change**	19 (-20, 58)	0.342	15 (-20, 51)	0.384
**Fat (g/day)**				
**16th-18th week**	-3 (-10, 4)	0.355	-2 (-8, 4)	0.559
**36th-38th week**	0 (-7, 8)	0.947	1 (-6, 7)	0.825
**Change**	3 (-4, 11)	0.385	3 (-4, 9)	0.466
**Carbohydrates (g/day)**				
**16th-18th week**	-1 (-4, 1)	0.300	-1 (-3, 2)	0.527
**36th-38th week**	0 (-3, 3)	0.990	0 (-2, 3)	0.746
**Change**	1 (-1, 4)	0.358	1 (-1, 3)	0.356
**Protein (g/day)**				
**16th-18th week**	-1 (-10, 7)	0.777	0 (-7, 8)	0.905
**36th-38th week**	1 (-9, 12)	0.807	1 (-8, 11)	0.775
**Change**	2 (-8, 12)	0.670	1 (-8, 10)	0.863
**Fibre (g/day)**				
**16th-18th week**	-9 (-29, 12)	0.403	1 (-18, 20)	0.887
**36th-38th week**	-3 (-25, 19)	0.764	-1 (-21, 19)	0.938
**Change**	4 (-19, 26)	0.755	-2 (-23, 19)	0.857

Effect sizes from regression models as estimated marginal mean difference (95% CI)

a) unadjusted model

b) linear mixed model adjusted for practice (random factor), maternal age and pre-pregnancy BMI, infant age at follow-up, birth weight and group
